# Supplementary material for: A sterol-binding pocket in iRhom1 underlies paralog-specific regulation of the sheddase ADAM17
Source: bioRxiv. 2026 May 1:2026.04.28.721421. Preprint. [Version 1] doi: 10.64898/2026.04.28.721421 (PMC13142363; doi:10.64898/2026.04.28.721421)
Supplement: Supplement 1 [file media-1.pdf]

Supplementary Information

A sterol-binding pocket in iRhom1 underlies paralog-specific regulation of the sheddase ADAM17

Fangfang Lu[1,3], Hongtu Zhao[2,4], Yaxin Dai[2], Chia-Hsueh Lee[2*] and Matthew Freeman[1*]

[1]Sir William Dunn School of Pathology, University of Oxford, South Parks Road, Oxford OX1 3RE, UK.

[2]Department of Structural Biology, St. Jude Children's Research Hospital, Memphis, TN, 38105, USA.

[3]Present address: Broad Institute of MIT and Harvard, Cambridge, MA, 02142, USA.

[4]Present address: Guangzhou National Laboratory, Guangdong, 510005, China.

[*]Correspondence should be addressed to C.-H.L. (chiahsueh.lee@stjude.org) or M.F. (matthew.freeman@path.ox.ac.uk).

Structure	ADAM17/iRhom1
PDB	12VV
EMDB	EMD-76788
**Data collection/ processing**	
Magnification	130,000x
Voltage (kV)	300
Pixel size (Å)	0.649
Defocus range (μm)	0.6-1.6
Electron exposure (e^-/Å^2)	62.3
Symmetry imposed	C1
Initial particles (No.)	~9.5 million
Final particles (No.)	335,665
Map resolution (Å)	2.50
FSC threshold	0.143
Map resolution range (Å)	40-2.2
**Refinement**	
Model Resolution (Å)	2.7
FSC threshold	0.5
Map sharpening B-factor (Å^2)	-72.8
Model composition	
Non-hydrogen atoms	9065
Protein residues	1139
Ligand	3
B-factors (Å^2)	
Protein	47.92
Ligand	38.26
R.m.s. deviations	
Bond lengths (Å)	0.005
Bond angles (°)	1.030
Validation	
MolProbity score	1.74
Clashscore	6.85
Rotamers outliers (%)	0.00
Ramachandran plot (%)	
Favored	94.79
Allowed	5.21
Outliers	0.00

Table S1. Cryo-EM data collection, processing, and refinement statistics

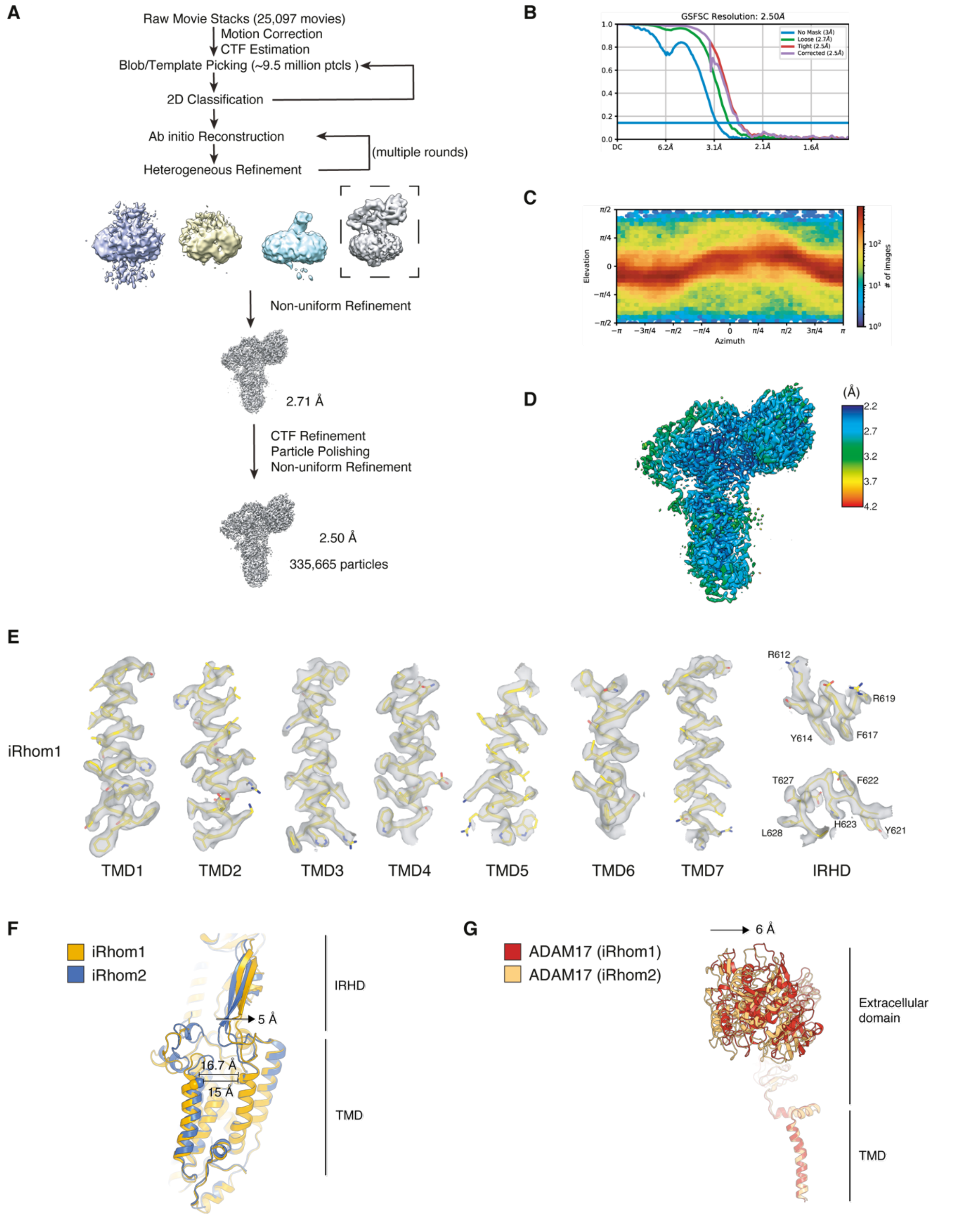

Figure S1. Cryo-EM processing, validation and structural comparison of the iRhom1/ADAM17 complex. (A) Cryo-EM data-processing workflow for the human iRhom1/ADAM17 complex. (B) Gold-standard Fourier shell correlation curve for the final iRhom1/ADAM17 reconstruction. (C) Angular distribution of particles for the final

3D reconstructions. (D) Local-resolution map of the iRhom1/ADAM17 complex, coloured according to local resolution. (E) Representative cryo-EM densities for iRhom1 transmembrane helices and the IRHD, showing the quality of the map in the modelled regions. (F) Structural superposition of the iRhom1 and iRhom2 transmembrane regions. iRhom1 and iRhom2 are shown in yellow and blue, respectively. Differences in the relative positions of the IRHD and transmembrane core are indicated. (G) Structural comparison of ADAM17 in the iRhom1/ADAM17 and iRhom2/ADAM17 complexes, showing a displacement of the ADAM17 extracellular domain between the two complexes.

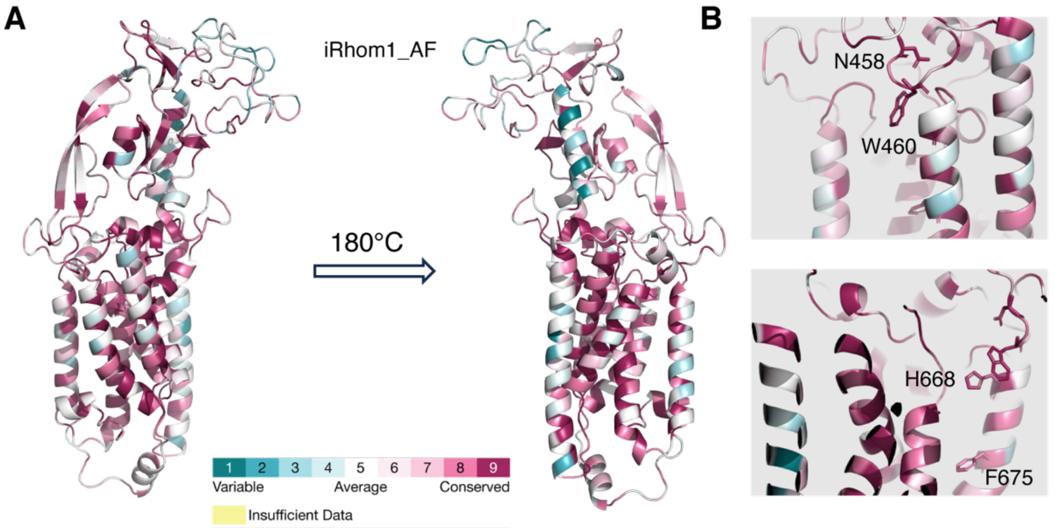

Figure S2. Conservation analysis of the iRhom1 sterol-binding pocket. (A) Human iRhom1 coloured according to the residue conservation analysed by ConSurf. AlphaFold model was used to show all residues of TMD and extracellular domain. (B) Close-up views of the iRhom1 sterol-binding pocket showing conservation score of residues N458, W460, H668 and F675 (side chains were shown as sticks).

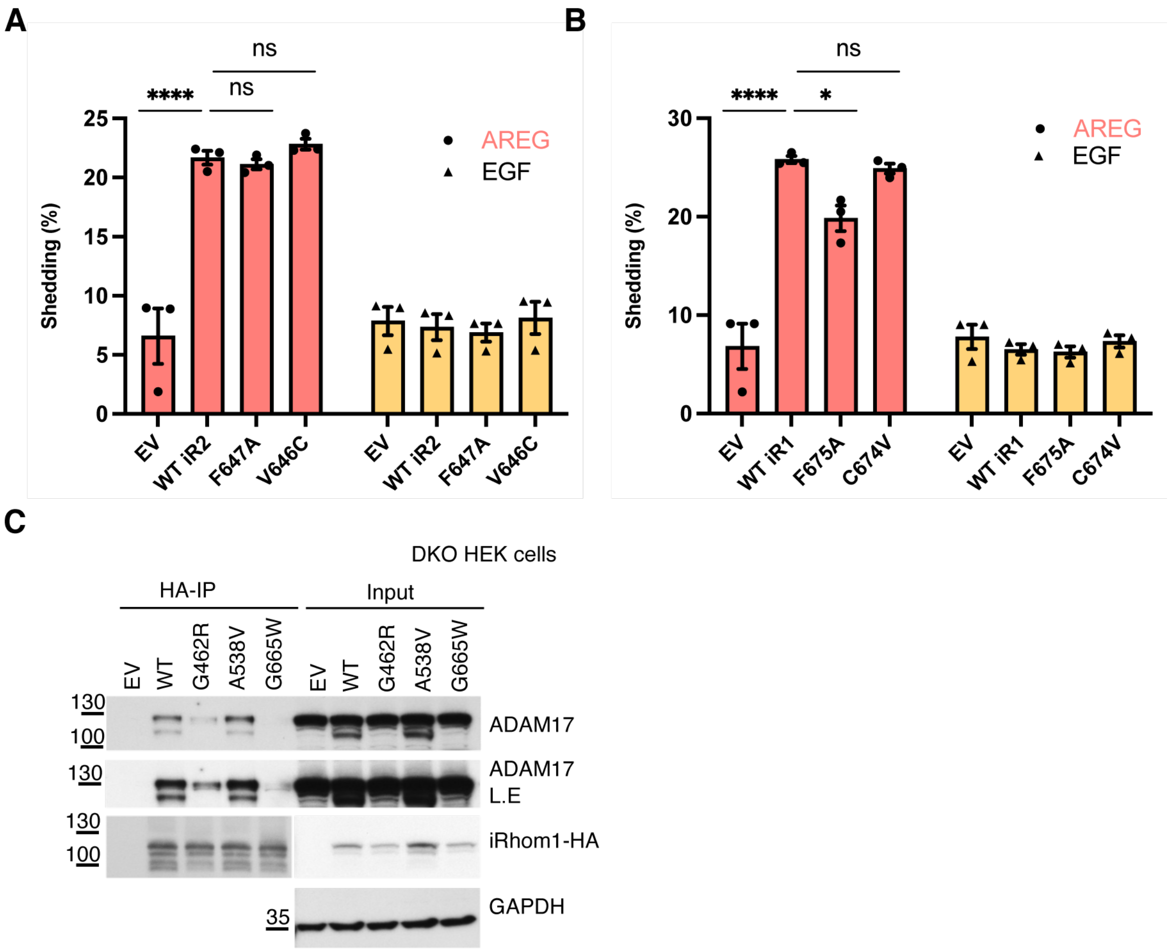

Figure S3. Additional functional analysis of iRhom1 and iRhom2 sterol-pocket and disease-associated variants. (A) ADAM17-dependent shedding assay in iRhom1/2 DKO cells expressing empty vector, wild-type iRhom2 isoform 2, or the indicated iRhom2 variants. Shedding of alkaline phosphatase-tagged AREG and EGF substrates was quantified. Data are shown as mean ± SD; ns = not significant, **** = $p < 0.0001$. (n=3, independent experiment). (B) ADAM17-dependent shedding assay in iRhom1/2 DKO cells expressing empty vector, wild-type iRhom1, or the indicated iRhom1 variants. Shedding of alkaline phosphatase-tagged AREG and EGF substrates was quantified. Data are shown as mean ± SEM; ns = not significant, * = $p < 0.05$, **** = $p < 0.0001$. (C) HA immunoprecipitation analysis in iRhom1/2 double-knockout HEK293T cells expressing empty vector, wild-type iRhom1, the control A538V variant, or the disease-associated G462R and G665W variants. Co-precipitated ADAM17 and iRhom1-HA were detected by immunoblotting; GAPDH served as a loading control.
